# Radical antegrade modular pancreatosplenectomy versus standard procedure in the treatment of left-sided pancreatic cancer: A systemic review and meta-analysis

**DOI:** 10.1186/s12893-017-0259-1

**Published:** 2017-06-05

**Authors:** Feng Cao, Jia Li, Ang Li, Fei Li

**Affiliations:** Department of General Surgery, Xuanwu Hospital, Capital Medical University, Beijing, 100053 People’s Republic of China

**Keywords:** Pancreatic body/tail cancer, Surgery, R0, Overall survival, Disease-free survival

## Abstract

**Background:**

Radical antegrade modular pancreatosplenectomy (RAMPS), first reported by Strasberg in 2003, has attracted increasing attention in the treatment of left-sided pancreatic cancer. The limited number of cases eligible for RAMPS makes it difficult to perform any prospective randomized trial of RAMPS versus the standard procedure. Therefore, we performed this systemic review and meta-analysis of the current data to clarify the role of the RAMPS procedure.

**Methods:**

A literature search was performed in electronic databases, including PubMed, Medline, Embase, CNKI and the Cochrane Library. Studies comparing RAMPS with the standard procedure were included in this meta-analysis. R0 resection rate, recurrence rate at the end of the follow-up, overall survival (OS) and disease-free survival (DFS) were measured as primary outcomes. Revman 5.3 was used to perform the analysis.

**Results:**

Six retrospective cohort studies with a total number of 378 patients were included in our analysis. Meta-analysis revealed that RAMPS was correlated with higher R0 resection rates [Odds Ratio (OR) 95% confidence interval (CI), 2.19 (1.16 ~ 4.13); *P* = 0.02] and successful harvest of more lymph nodes [weighted mean difference (WMD) 95% CI, 7.06 (4.52 ~ 9.60); *P* < 0.01] compared with the standard procedure. However, no statistically significant difference was found between the procedures with respect to recurrence rates [OR 95% CI, 0.66 (0.40 ~ 1.09); *P* = 0.10], OS [Hazard ratio (HR) 95% CI, 0.65 (0.42 ~ 1.00); *P* = 0.05] or DFS [HR 95% CI, 1.02 (0.62 ~ 1.68); *P* = 0.93].

**Conclusions:**

RAMPS is safe and oncologically superior to the standard procedure for the treatment of left-sided pancreatic cancer. However, high-grade evidence will be necessary to confirm the potential survival benefits of RAMPS.

**Electronic supplementary material:**

The online version of this article (doi:10.1186/s12893-017-0259-1) contains supplementary material, which is available to authorized users.

## Background

Distal pancreatectomy is the standard surgical approach for left-sided pancreatic cancer. However, the long-term survival of these patients remains unsatisfactory, with a median survival time of 10–28 months and a 5-year overall survival of 6–30% [1–5]. In recent years, new surgical approaches for resectable or borderline resectable pancreatic cancer, including the artery-first approach [6–9], superior mesenteric vein/portal vein resection and reconstruction [10–13], intraoperative radiotherapy [14, 15] and preoperative chemo-radiotherapy [16–18], have been increasingly applied to pancreaticoduodenectomy to achieve R0 resection for carcinomas of the head of the pancreas. Despite the highly aggressive nature of the disease and early regional lymph node metastasis, adenocarcinomas of the body and tail of the pancreas have attracted significantly less clinical attention. However, in 2003, Strasberg described a new distal pancreatectomy technique, termed radical antegrade modular pancreatosplenectomy (RAMPS), to achieve negative posterior resection margins and to completely remove the N1 lymph nodes [19]. In the past decade, the RAMPS procedure has been increasingly applied, particularly in Japan and Korea [20–24]. However, the number of patients eligible for RAMPS is too small to consider any prospective randomized trial of RAMPS versus the standard procedure. Therefore, systemic review and meta-analysis of the current retrospective data comparing RAMPS and the standard procedure are necessary and useful to clarify the role the RAMPS in the treatment of left-sided pancreatic cancer.

## Methods

### Search strategy and selection of trials

A computerized search was performed in July 2016 using the following terms: “radical antegrade modular pancreatosplenectomy” or “RAMPS”. The following electronic databases were included: PubMed, Medline, Embase, CNKI and the Cochrane Library. The reference list of selected articles was also reviewed.

Randomized controlled trials (RCTs) and retrospective cohort studies (RCSs) comparing RAMPS and the standard procedure for the treatment of left-sided pancreatic cancer were included in this systemic review and meta-analysis. There were no limitations with respect to language or date. Case reports, review articles and letters were not included, and studies without any major postoperative outcomes were excluded from the search results.

### Data extraction and quality assessment

Two reviewers (FC and JL) independently considered the eligibility of potential titles and extracted the data. Discrepancies were resolved by mutual discussion. Inclusion and exclusion criteria, country and year of publication, study type, number of patients operated on with each technique and the general characteristics of patients (age, gender, perioperative outcome and postoperative results) were extracted. The risk of bias for the trials enrolled in the meta-analysis was evaluated according to the Cochrane Handbook for Systematic Reviews of Interventions, and the quality of the non-randomized studies was assessed using the criteria suggested by the Newcastle-Ottawa quality assessment (NOS) tool [25]. This scale rates studies on a scale of one to nine, with nine representing the highest methodological quality, a NOS score of 7 or above considered high quality, and a NOS score of 3 or below considered low quality.

### Outcome measurements

The primary outcomes of this study were R0 resection rate, overall survival (OS) and disease-free survival (DFS); secondary outcomes included recurrence rate at the end of the follow-up, postoperative complication rate, intraoperative blood loss, operative time, the number of lymph nodes harvested, combined resection rate and duration of hospital stay.

### Statistical analysis

Meta-analysis was performed according to recommendations from the Cochrane Collaboration. Hazard ratios (HRs) and 95% confidence intervals (CIs), derived from values reported explicitly in the published studies or calculated from the Kaplan-Meier survival curve using the methods reported by Tierney and colleagues [26], were combined to measure the survival rates. Odds ratios (ORs) and weighted mean differences (WMDs) were used to measure dichotomous and continuous data, respectively. A combined HR/OR >1 and WMD > 0 indicated poor outcomes for patients in the RAMPS group (except R0 resection and the number of lymph node harvested). Heterogeneity was evaluated using the Chi-square test, and a *P* value less than 0.1 was considered statistically significant. The fixed effect model was used throughout the analysis unless significant heterogeneity was detected. Funnel plot and Egger’s test were used to investigate the publication bias. Analysis was performed using the Review Manager version 5.3 (Cochrane Collaboration, Software Update, Oxford, UK) and STATA/SE software version 12.0 (STATA Corporation, College Station, TX, USA).

## Results

### Characteristics of the trials

**Fig. 1:**
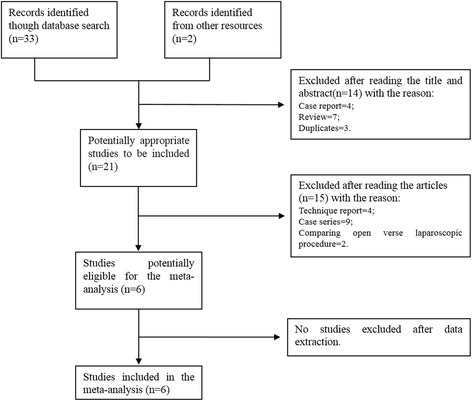
Flow diagram of studies included in the meta-analysis

Six retrospective trials that met the inclusion criteria were included in the meta-analysis for a total of 378 patients, including 152 patients undergoing RAMPS and 226 patients undergoing the standard procedure [20, 24, 27–30]. Figure 1 summarized the study flow. The patient characteristics and surgical outcomes of the included trials are summarized in Tables 1 and 2. No RCTs had been published at the time of our search. The risk of bias was evaluated by the Newcastle-Ottawa scale. Three studies earned a score of 7 or more and were considered high quality [20, 24, 30] (Additional file 1: Table S1). Outcomes may have been influenced by allocation bias in all studies for patients who underwent RAMPS or the standard procedure. Furthermore, the follow-up method was unclear in all of the studies.

**Table 1:** Characteristics of patients of included studies

References	Country	Published Year	Group	No. of patients	Age(year)	M/F	Tumor size (cm)	CA19–9 level (U/ml)	T3 + T4	N+	Well differentiation	Quality of study^a^
Latorre [28]	Italy	2013	RAMPS	8	61	5/3	5.1 ± 1.9	NA	NA	NA	3	6
			Standard	17	60	11/6		NA	NA	NA		
Park [24]	Korea	2014	RAMPS	38	62.17 (40–75)	23/15	3.1 (2–8.0)	18.2 (3.0–82.1)	37	22	4	7
			Standard	54	61.25 (37–79)	35/19	3.8 (1–11)	15.7 (4.4–148.5)	51	22	3	
Trottman [27]	USA	2014	RAMPS	6	NA	NA	NA	NA	NA	NA	NA	3
			Standard	20	NA	NA	NA	NA	NA	NA	NA	
Abe [20]	Japan	2016	RAMPS	53	68.6 ± 10.7	1.40:1	NA	136.4 ± 291.0	38	28	3	7
			Standard	40	65.2 ± 8.6	2.63:1	NA	390.4 ± 1157.1	34	26	7	
Xu [29]	China	2016	RAMPS	21	62 ± 11	11/10	5(4.3–6.6)	70.2(20.7–594.2)	21	11	NA	6
			Standard	78	63 ± 9	41/37	3.8(3.0–5.0)	158.7(35.6–692.2)	63	26	NA	
Kim [30]	Korea	2016	RAMPS	30	63.7 ± 8.2	13/17	4.6 ± 1.6	NA	25	14	3	8
			Standard	19	62.1 ± 8.5	7/12	4.5 ± 1.5	NA	13	6	2	

*M/F* male/female, *NA* not available. ^a^according to Newcastle-Ottawa quality assessment scale

**Table 2:** Surgical outcomes of patients of included studies

References	Group	Intraoperative blood loss(ml)	Operative time (min)	Lymph node harvested	Complication	R0 resection	Combined resection	Hospital stay (days)	Recurrence	HR(95% CI) for DFS	HR(95% CI) for OS
Latorre [28]	RAMPS	342	315	20.7 ± 8.9	2	7(87.5%)	4	12.1	NA	1.32 (0.45–3.92)	1.26 (0.45–3.57)
	Standard	369	265	16.2 ± 4.2	5	15(88.2%)		9.9	NA		
Park [24]	RAMPS	325 (50–3400)	210 (125–480)	14(5–52)	7	34(89.5%)	15	11.5(7–32)	25(65.6%)	NA	0.56 (0.32–0.98)
	Standard	400 (50–3300)	185 (80–390)	9(1–36)	12	46(85.2%)	11	10.7(6–42)	35(64.8%)		
Trottman [27]	RAMPS	500.0 ± 260.8	300.0 ± 87.0	11.2 ± 6.0	3	6(100%)	NA	7.7 ± 3.0	NA	NA	NA
	Standard	581.3 ± 559.2	295.3 ± 83.8	4.3 ± 5.4	12	19(95%)	NA	6.9 ± 1.4	NA		
Abe [20]	RAMPS	485.4 ± 63.3	267.3 ± 11.5	28.4 ± 11.6	19	48(90.6%)	8	35.7 ± 19.6	32(60.4%)	0.96 (0.54–1.71)	0.66 (0.21–2.11)
	Standard	682.3 ± 72.8	339.4 ± 13.2	20.7 ± 10.1	14	27(67.5%)	5	26.7 ± 25.5	30(75.0%)		
Xu^a^ [29]	RAMPS	400(350–650)	235(180–278)	NA	16	19(90.5%)	13	15(13–23)	6(33.3%)	NA	NA
	Standard	225(200–400)	180(130–210)	NA	48	71(91.0%)	10	12(10–16)	31(45.6%)		
Kim^b^ [30]	RAMPS	300 ± 220	277.8 ± 55.6	21.5 ± 8.3	14	22(84.6%)	NA	6.4 ± 4.3	8(30.8%)	0.90 (0.08–9.92)	0.48 (0.13–1.83)
	Standard	260 ± 180	253.3 ± 41.0	13.7 ± 7.4	8	11(64.%7)	NA	8.2 ± 3.3	8(47.1%)		

*NA* not available. ^a^Three and 10 patients in RAMPS and standard group were loss of follow-up (median 18 months, range 5–37 months) in the study period. ^b^Two patients who had neuroendocrine carcinoma and two who had metastatic renal cell carcinoma in RAMPS group and two patients who had neuroendocrine carcinoma in standard group were excluded from the analyses of R0 and recurrence rate

### Meta-analysis results

#### Primary outcome

##### R0 resection rate

**Fig. 2:**
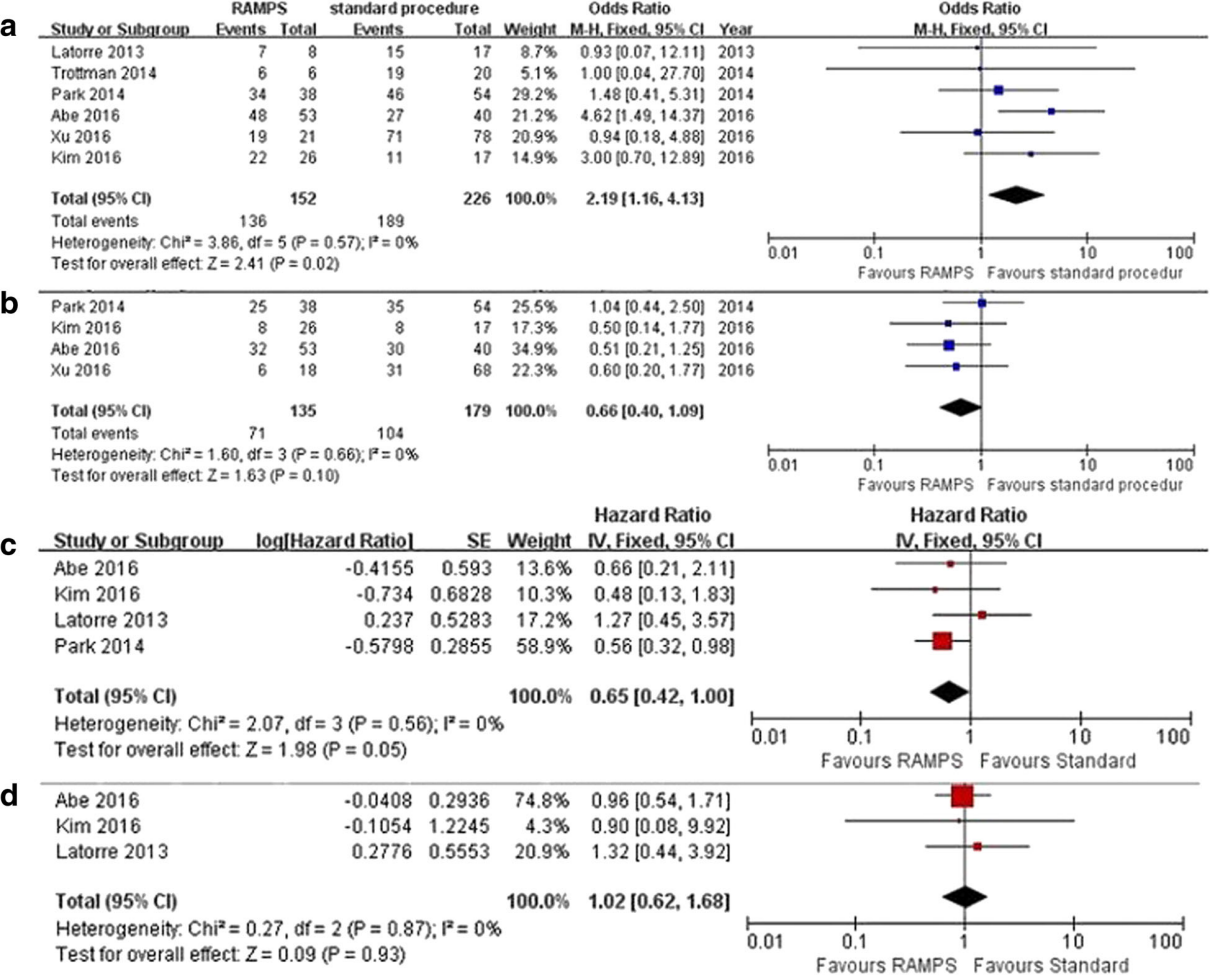
Meta-analysis for results **a** R0 resection rate, **b** recurrence rate, **c** overall survival (OS), **d** disease-free survival (DFS)

All of the included studies reported R0 resection rates [20, 24, 27–30]. The R0 resection rate was 89.5% (136/152) in the RAMPS group and 83.6% (189/226) in the standard surgery group. The overall analysis revealed that the R0 resection rate was significantly higher in the RAMPS group than in the standard surgery group [OR 95% CI, 2.19 (1.16 ~ 4.13); *P* = 0.02] (Fig. 2a). Heterogeneity was not detected (*P* = 0.57, I^2^ = 0%), and the fixed-effects model was used.

##### Recurrence rate at the end of the follow-up

The four large studies reported recurrence rates at the end of the follow-up [20, 24, 29, 30]: 52.6% and 58.1% in the RAMPS and standard groups, respectively. Overall analysis revealed that there was no statistically significant difference between the groups with respect to the recurrence rate [OR 95% CI, 0.66 (0.40 ~ 1.09); *P* = 0.10] (Fig. 2b).

##### Overall survival

Four of the included studies reported the overall survival rate [20, 24, 28, 30]. Heterogeneity was not detected among these studies (*P* = 0.56, I^2^ = 0%), and the fixed-effected model was used. Overall analysis revealed no significant difference between the RAMPS and standard surgery groups [HR 95% CI, 0.65 (0.42 ~ 1.00); *P* = 0.05] (Fig. 2c).

##### Disease-free survival

Three studies reported disease-free survival rates [20, 28, 30]. No significant difference was found when comparing RAMPS with the standard procedure [HR 95% CI, 1.02 (0.62 ~ 1.68); *P* = 0.93] using a fixed-effect model (*P* = 0.87, I^2^ = 0%) (Fig. 2d).

#### Secondary outcomes

Meta-analysis results for secondary outcomes, including postoperative complication rate, intraoperative blood loss, operative time, number of lymph nodes harvested, combined resection rate and duration of hospital stay, are summarized in Table 3. The number of lymph nodes harvested in the RAMPS group was significantly greater than that in the standard operation group [WMD 95% CI, 7.06 (4.52 ~ 9.60); *P* < 0.01] without increased intraoperative blood loss [−85.11 (−278.08 ~ 107.85); *P* = 0.39]. Despite the tendency toward higher combined resection rates [OR 95% CI, 3.30 (1.00 ~ 10.93); *P* = 0.05], the incidence of complications in the RAMPS group did not increase [OR 95% CI, 0.94 (0.56 ~ 1.59); *P* = 0.83]. There were no statistically significant differences between the groups with respect to operative time or duration of hospital stay (Additional file 2: Figure S1, Additional file 3: Figure S2, Additional file 4: Figure S3, Additional file 5: Figure S4, Additional file 6: Figure S5, Additional file 7: Figure S6).

**Table 3:** Secondly results of meta-analysis for RAMPS verse standard procedure in treatment of left-sided pancreatic cancer

Outcome	Ref. included	No. of patients with RAMPS vs no standard	Heterogeneity Chi-square test	Model used	OR or Mean difference	95% CI	*P* value
Intraoperative blood loss(ml)	[20, 27, 30]	89 vs 79	*P* < 0.01; I^2^ = 88%	Random effect	−85.11	−278.08-107.85	0.39
Operating time (min)	[20, 27, 30]	89 vs 79	*P* < 0.01; I^2^ = 96%	Random effect	−16.81	−95.19-61.57	0.67
Lymph node harvested	[20, 27, 28]	93 vs 94	*P* = 0.86; I^2^ = 0%	Fixed effect	7.06	4.52–9.60	<0.01
Complication	[20, 24, 27–29]	135 vs 150	*P* = 0.97; I^2^ = 0%	Fixed effect	0.94	0.56–1.59	0.83
Combined resection	[20, 24, 29]	112 vs 172	*P* = 0.02; I^2^ = 73%	Random effect	3.30	1.00–10.93	0.05
Hospital stay (days)	[20, 27, 30]	89 vs 79	*P* = 0.04; I^2^ = 68%	Random effect	0.49	−2.97-3.94	0.78

*OR* odds ratio, *CI* confidence intervals

#### Sensitivity analyses

To test the stability of the overall meta-analysis results, sensitivity analyses were conducted by excluding low quality studies [27–29]. The results of these analyses revealed no significant differences when compared with the former estimates (Additional file 8: Table S2).

### Publication bias

Funnel plots for primary results were drawn to assess potential publication bias (Additional file 9: Figure S7). All of the plots were symmetrical, suggesting no reporting bias among the studies. Egger’s test for OS (*t* = 0.51, *P* = 0.659) and DFS (*t* = 0.33, *P* = 0.795) revealed no publication bias.

## Discussion

The RAMPS procedure, first reported in 2003, was designed to establish an operation with oncologic safety both with respect to the dissection planes used to achieve negative margins as well as the extent of lymph node dissection, thereby improving patient outcomes. According to the original paper by Strasberg, if the tumour did not penetrate the posterior capsule of the pancreas on preoperative CT scans, the resection plane lay just behind the anterior renal fascia, and anterior RAMPS was performed; otherwise, posterior RAMPS was applied, and the left adrenal gland and Gerota fascia were removed [19]. Deep resection is performed because tumours can spread microscopically beyond their radiographically visible or palpable margins. The systemic review of descriptive studies concerning the RAMPS procedure for the treatment of left-sided pancreatic cancer is summarized in Table 4. R0 resection was achieved in 77–100% of patients after RAMPS, and an R0 rate > 85% was observed in most case series. In this meta-analysis, we found that the R0 resection rate was significantly higher in the RAMPS group than in the standard surgery group [89.5% vs 83.6%, OR 95% CI, 2.19 (1.16 ~ 4.13); *P* = 0.02]. However, the combined resection rates were comparable between the RAMPS and standard groups [OR 95% CI, 3.30 (1.00 ~ 10.93); *P* = 0.05], which might be attributable to the low rate of posterior RAMPS procedures in present practices [24, 31, 32].

**Table 4:** Systemic review of descriptive studies about RAMPS procedure in treatment of left-sided pancreatic cancer

Reference	Year	No. of patients	A/P RAMPS	Tumor size (cm)	N+(%)	R0(%)	Lymph Node harvested	Median follow-up time(months)	Recurrence rate (%)	Median survival time (months)	5-year overall survival (%)
Strasberg [19]	2003	10	6/4	4(2–15)	NA	90	1–28	NA	3(30.0%)	NA	NA
Strasberg [32]	2007	23	15/8	5.1 ± 2.6	48	87	14.3 ± 7.8	17 for alive	11(47.8%)	NA	NA
Kang^a^ [41]	2010	5	5/0	2.4 ± 0.7	20	100	8.2 ± 5.9	13(4–21)	1(20%)	NA	NA
Ikegami [42]	2011	6	3/3	3.0 ± 0.9	NA	100	NA	NA	NA	NA	NA
Mitchem [43]	2012	47	32/15	4.4 ± 2.1	55	80.1	18.0 ± 11.7	26.4 for alive	27(57.4%)	25.9	35.5
Chang [44]	2012	24	19/5	4.09 ± 2.15	70.8	91.7	20.92 ± 11.24	20.06	21(87.5%)	18.2	NA
Kim [45]	2013	12	12/0	2(0.8–4.0)	50	NA	17(5–29)	NA	NA	NA	NA
Rosso [46]	2013	10	1/9	4.65(1.0–8.0)	70	90	17(13–95)	19.1 ± 10.1	NA	20.5%	NA
Lee^b^ [39]	2014	12	12/0	2.75 ± 1.32	25	100	10.5 ± 7.14	39	5(41.7%)	60.0	55.6
Kitagawa^c^ [38]	2014	24	19/5	3.5 ± 1.4	54.2	88	28 ± 12	52 for alive	10(41.7%)	NA	53
Kawabata^d^ [37]	2015	11	NA	3.35(1.9–5.5)	91	77	26(9–80)	12.4(3.5–16.4)	1(9.1%)	NA	NA
Murakawa [12]	2015	49	NA	0.5–8.3	55	83.7	15	41.4	30(61.2%)	22.6	27
Grossman [31]	2016	78	56/22	4.71	47	85	20 ± 12.2	20.6 (0.3–145.3)	49(62.8%)	24.6	25.1

*A/P* anterior/posterior, *NA* not available. ^a^laparoscopic or robot-assisted anterior RAMPS; ^b^laparoscopic modified anterior RAMPS in well-selected patients with Yonsei criteria; ^c^modified RAMPS; ^d^RAMPS with artery-first approach

Lymph node metastasis has been reported to be an independent prognostic risk factor for resected left-sided pancreatic cancer [33, 34]. The extent of lymph node dissection is one of the key points of pancreatosplenectomy. However, guidelines from Eastern and Western countries differ significantly. In the RAMPS procedure, the lymph nodes along the superior and inferior borders of the left-sided pancreas (No. 10, 11, and 18 according to Japanese classification), the celiac lymph nodes (No. 9) and the nodes along the front and left side of the superior mesenteric artery (No. 14p, 14d) are considered N1 lymph nodes and are completely removed; in the standard operation, only lymph nodes No. 10, 11, and 18 are resected [35]. Therefore, in this meta-analysis, we found that the number of lymph nodes harvested in the RAMPS procedure was significantly greater than in the standard operation [WMD 95% CI, 7.06 (4.52–9.60); *P* < 0.01]. Compared with the standard operation, the RAMPS procedure is reported to require greater technical skill for extensive resection as well as longer operating times [24, 28]. However, these differences were not detected in our meta-analysis [WMD 95% CI, −16.81 (−95.19–61.57); *P* = 0.67]. Additionally, RAMPS procedures were not correlated with longer hospital stays [WMD 95% CI, 0.49 (−2.97–3.94); *P* = 0.78]. These findings may be influenced by a recent study with a large volume of patients and more experienced surgeons.

Improving the survival of patients with resectable or borderline resectable tumours is the major aim of the RAMPS procedure. The 5-year survival rate after RAMPS ranged from 25.1% to 55.6% (Table 4). In a recent study, when comparing RAMPS and the standard procedure, RAMPS exhibited a greater tendency towards improvement of median survival times relative to the standard procedure (47 vs 34 months, *P* = 0.192), but no significant differences in the recurrence rates were detected (66.6 vs 75.0%; *P* = 0.1386) [20]. In the study by Park, the 5-year overall survival rate was 40.1% in RAMPS patients and 12.0% in the standard group (*p* = 0.014). However, by multivariate analysis, adjuvant chemoradiotherapy but not RAMPS reached statistical significance with respect to overall survival [24]. In the present study, no favourable overall survival outcomes were detected when comparing RAMPS with the standard procedure. The recurrence rate after RAMPS did not decrease (65.7% vs 64.8%, *P* = 0.482), which was consistent with our meta-analysis [OR 95% CI, 0.66 (0.40 ~ 1.09); *P* = 0.10] and led to similar DFS rates in the two groups [OR 95% CI, 1.02 (0.62 ~ 1.68); *P* = 0.93]. With respect to recurrence, we believed that it is important to differentiate local recurrence from systemic recurrence. RAMPS increased the R0 resection rate and theoretically may decrease local recurrence. Unfortunately, few studies reported the local recurrence rate. In these studies, systemic recurrence alone, such as liver, lung and peritoneum, was reported most often, and the local recurrence rate did not decrease significantly after RAMPS [20, 31].

Recently, a modified RAMPS procedure including a superior mesenteric artery (SMA)-first approach has been attempted [22, 36–38]. The artery-first approach, initially designed for the early determination of cancer resectability during pancreatoduodenectomy, is now applied in the RAMPS procedure. As described by Strasberg, dissection of the SMA is performed after transection of the pancreas or wide detachment of the distal pancreas and spleen, which may reach the point of no return. However, carcinoma of the pancreatic body and tail exhibits high aggressive potential, and the celiac axis (CA) and SMA are often involved. Although left-sided pancreatic cancer with CA invasion can be treated by distal pancreatectomy combined with celiac axis resection (DP-CAR), SMA encroachment usually indicates that the tumour is a late-stage lesion and may be completely unresectable. SMA-first RAMPS provides an opportunity to determine resectability before pancreas transection. Dissection further along the aorta and exposure of the left renal vein and the left adrenal gland can help prepare the correct RAMPS dissection plane in advance. When the renal vein is reached, the surgeon can accurately assess the extent of tumour penetration to help decide whether anterior or posterior RAMPS is optimal. Data from Japan has demonstrated the safety and reliability of this procedure even in borderline resectable tumours [22, 36, 37].

Laparoscopic or robotic RAMPS operations have also been performed with satisfactory oncological results and survival outcomes [39–41]. However, this procedure is limited to highly selective cases. According to the Yonsei criteria developed by Lee, only patients meeting the following characteristics can be treated with minimally invasive RAMPS: (1) tumour confined to the pancreas, (2) intact fascial layer between the distal pancreas and the left adrenal gland and kidney, and (3) tumour located more than 1–2 cm from the celiac axis [39].

An important limitation of this review is the small number of included studies and cases. In addition, the nature of the included retrospective studies may lead to allocation and publication biases and could distort the conclusions of this review. However, this study represents the initial attempt to perform a systemic review and meta-analysis of RAMPS versus the standard procedure in the treatment of left-sided pancreatic cancer. Our systematic review and meta-analysis presents evidence to suggest that RAMPS is the optimal procedure to increase R0 resection rates but has no increased benefit with respect to tumour recurrence or patient survival.

## Conclusion

The RAMPS procedure for the treatment of left-sided pancreatic cancer can achieve higher rates of R0 resection without increasing complication rates compared with the standard procedure. However, high-grade evidence is required before any conclusions may be made concerning the survival benefit of RAMPS.

## Additional files

Additional file 1: Table S1. Risk of bias in the included retrospective cohort studies (by the Newcastle–Ottawa quality assessment tool). (DOCX 12 kb)

Additional file 2: Figure S1. Meta-analysis for lymph node harvested showed significantly greater in RAMPS group. (PNG 9 kb)

Additional file 3: Figure S2. Meta-analysis revealed compared result for intraoperative blood loss. (PNG 10 kb)

Additional file 4: Figure S3. Meta-analysis for combined resection rate. RAMPS procedure did not combined resection rate. (PNG 9 kb)

Additional file 5: Figure S4. Meta-analysis revealed that RAMPS did not increase the complication. (PNG 10 kb)

Additional file 6: Figure S5. Meta-analysis for operation time showed compared result between RAMPS and standard procedure. (PNG 10 kb)

Additional file 7: Figure S6. Meta-analysis revealed similar hospital stay in RAMPS and standard procedure. (PNG 9 kb)

Additional file 8: Table S2. Results of sensitivity analyses which revealed no significant differences when compared with main analyses. (DOCX 13 kb)

Additional file 9: Figure S7. Funnel plots for (a) R0 resection, (b) recurrence, (c) OS and (d) DFS revealed no publication bias. (PNG 182 kb)
