# Supplementary material for: Radical antegrade modular pancreatosplenectomy versus standard procedure in the treatment of left-sided pancreatic cancer: A systemic review and meta-analysis
Source: BMC Surg. 2017 Jun 5;17:67. doi: 10.1186/s12893-017-0259-1 (PMC5460359; doi:10.1186/s12893-017-0259-1)
Supplement: Supplementary file 1 — Risk of bias in the included retrospective cohort studies (by the Newcastle–Ottawa quality assessment tool). (DOCX 12 kb) [file 12893_2017_259_MOESM1_ESM.docx]

Table S1 Risk of bias in the included retrospective cohort studies (by the Newcastle–Ottawa quality assessment tool)

| **References** | **Selection/4** | **Comparability/2** | **Outcome/3** | **Total score** |
| --- | --- | --- | --- | --- |
| Latorre | 3 | 1 | 2 | 6 |
| Park | 3 | 2 | 2 | 7 |
| Trottman | 3 | 0 | 0 | 3 |
| Abe | 3 | 2 | 2 | 7 |
| Xu | 3 | 1 | 2 | 6 |
| Kim | 3 | 2 | 3 | 8 |
