# Supplementary figures and images for: Radical antegrade modular pancreatosplenectomy versus standard procedure in the treatment of left-sided pancreatic cancer: A systemic review and meta-analysis
Source: BMC Surg. 2017 Jun 5;17:67. doi: 10.1186/s12893-017-0259-1 (PMC5460359; doi:10.1186/s12893-017-0259-1)

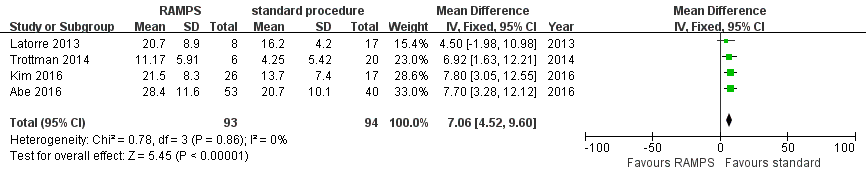

Supplement: Supplementary file 2 — Meta-analysis for lymph node harvested showed significantly greater in RAMPS group. (PNG 9 kb) [file 12893_2017_259_MOESM2_ESM.png]

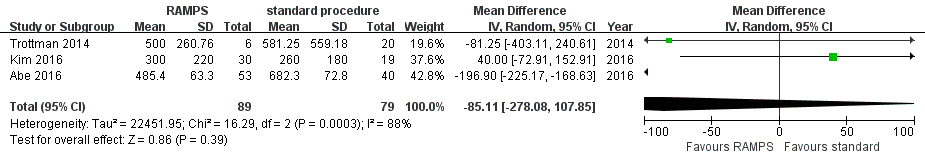

Supplement: Supplementary file 3 — Meta-analysis revealed compared result for intraoperative blood loss. (PNG 10 kb) [file 12893_2017_259_MOESM3_ESM.png]

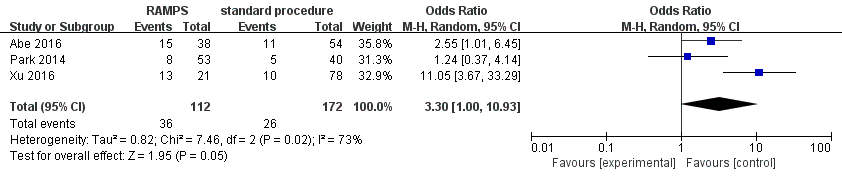

Supplement: Supplementary file 4 — Meta-analysis for combined resection rate. RAMPS procedure did not combined resection rate. (PNG 9 kb) [file 12893_2017_259_MOESM4_ESM.png]

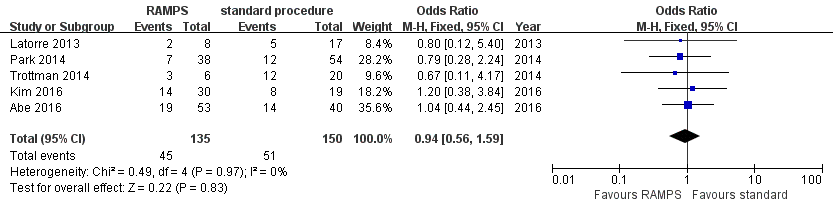

Supplement: Supplementary file 5 — Meta-analysis revealed that RAMPS did not increase the complication. (PNG 10 kb) [file 12893_2017_259_MOESM5_ESM.png]

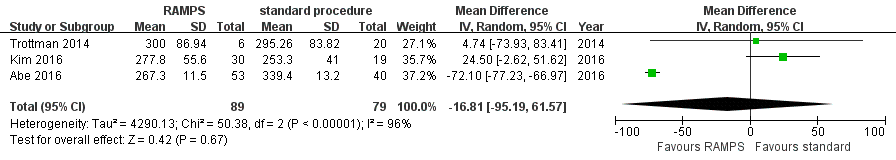

Supplement: Supplementary file 6 — Meta-analysis for operation time showed compared result between RAMPS and standard procedure. (PNG 10 kb) [file 12893_2017_259_MOESM6_ESM.png]

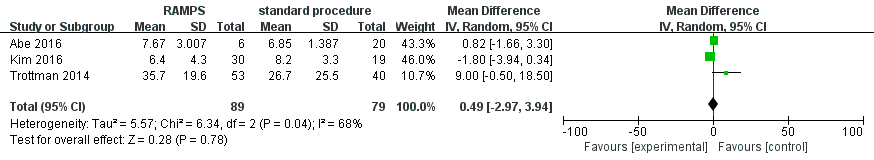

Supplement: Supplementary file 7 — Meta-analysis revealed similar hospital stay in RAMPS and standard procedure. (PNG 9 kb) [file 12893_2017_259_MOESM7_ESM.png]

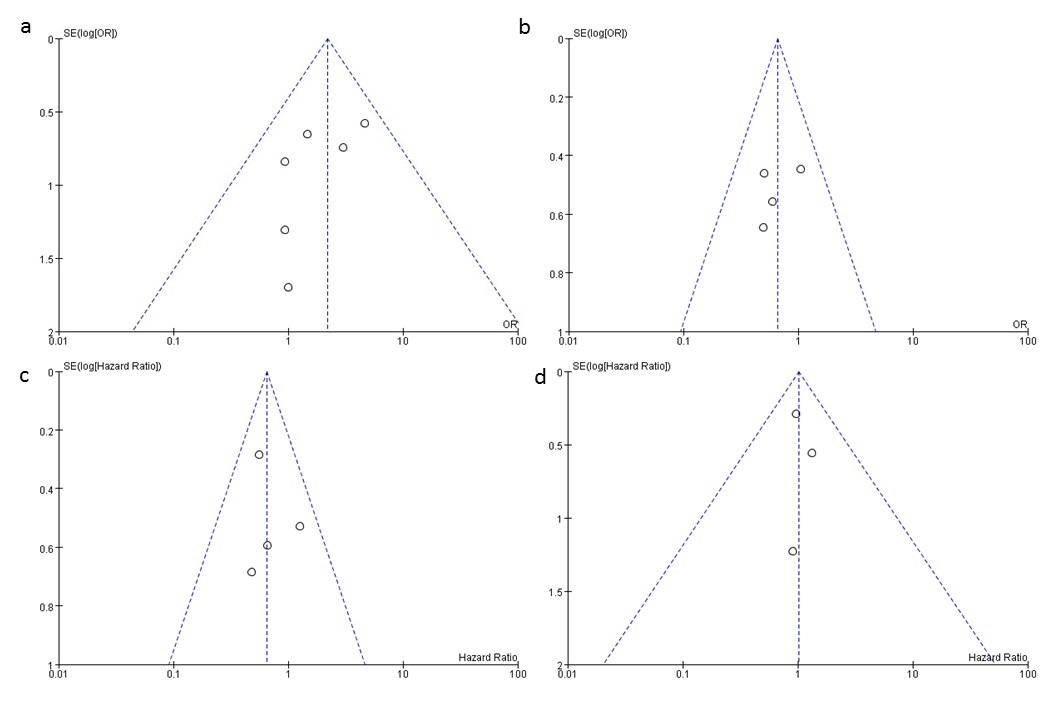

Supplement: Supplementary file 9 — Funnel plots for (a) R0 resection, (b) recurrence, (c) OS and (d) DFS revealed no publication bias. (PNG 182 kb) [file 12893_2017_259_MOESM9_ESM.png]
