# Supplementary material for: Radical antegrade modular pancreatosplenectomy versus standard procedure in the treatment of left-sided pancreatic cancer: A systemic review and meta-analysis
Source: BMC Surg. 2017 Jun 5;17:67. doi: 10.1186/s12893-017-0259-1 (PMC5460359; doi:10.1186/s12893-017-0259-1)
Supplement: Supplementary file 8 — Results of sensitivity analyses which revealed no significant differences when compared with main analyses. (DOCX 13 kb) [file 12893_2017_259_MOESM8_ESM.docx]

Table S2 Results of sensitivity analyses

| **Outcome** | **Ref. included** | **No. of patients with RAMPS *vs* no standard** | **Heterogeneity Chi-square test** | **Model used** | **OR or Mean difference** | **95%CI** | **P value** |
| --- | --- | --- | --- | --- | --- | --- | --- |
| R0 resection rate^*^ | 20,24,28 | 117 vs 111 | P=0.42；I^2^=0% | Fixed effect | 2.85 | 1.37-5.90 | 0.005 |
| Recurrence rate^*^ | 20,24,28 | 117 VS 111 | P=0.46；I^2^=0% | Fixed effect | 0.68 | 0.39-1.19 | 0.18 |
| Intraoperative blood loss(ml) | 20,28 | 83 *vs* 59 | P＜0.01; 94% | Random effect | -85.02 | -316.82-146.79 | 0.47 |
| Operating time (min) | 20,28 | 83 *vs* 59 | P＜0.01; I^2^=98% | Random effect | -24.76 | -119.40-69.89 | 0.61 |
| Lymph node harvested^*^ | 20,28 | 79 *vs* 57 | P=0.98; I^2^=0% | Fixed effect | 7.75 | 4.51-10.98 | ＜0.01 |
| Complication | 20,24,28 | 121 *vs* 113 | P=0.86; I^2^=0% | Fixed effect | 0.99 | 0.56-1.75 | 0.97 |
| Combined resection | 20,24 | 91 *vs* 94 | P=0.35; I^2^=0% | Fixed effect | 1.95 | .94-4.06 | 0.07 |
| Hospital stay (days) | 20,28 | 83 *vs* 59 | P=0.03; I^2^=79% | Random effect | 2.57 | -7.82-12.96 | 0.63 |

* Four patients in RAMPS group and 2 patients in standard group from Ref. 28 were excluded from the analyses.
